# Naringenin ameliorates cytotoxic effects of bisphenol A on mouse Sertoli cells by suppressing oxidative stress and modulating mitophagy: An experimental study

**DOI:** 10.18502/ijrm.v22i3.16166

**Published:** 2024-05-15

**Authors:** Layasadat Khorsandi, Abbas Heidari-Moghadam, Elham Younesi, Mohammad Javad Khodayar, Yousef Asadi-Fard

**Affiliations:** ^1^Cellular and Molecular Research Center, Medical Basic Sciences Research Institute, Ahvaz Jundishapur University of Medical Sciences, Ahvaz, Iran.; ^2^Department of Anatomical Sciences, Faculty of Medicine, Ahvaz Jundishapur University of Medical Sciences, Ahvaz, Iran.; ^3^Department of Anatomical Sciences, Faculty of Medicine, Dezful University of Medical Sciences, Dezful, Iran.; ^4^Toxicology Research Center, Medical Basic Sciences Research Institute, Ahvaz Jundishapur University of Medical Sciences, Ahvaz, Iran.; ^5^Department of Anatomy, School of Medicine, Arak University of Medical Sciences, Arak, Iran.

**Keywords:** Mitophagy, Naringenin, Sertoli cells, Bisphenol A, Reactive oxygen species, Pink1, Parkin.

## Abstract

**Background:**

Bisphenol A (BPA), an endocrine-disrupting agent, is widely used as polycarbonate plastics for producing food containers. BPA exposure at environmentally relevant concentrations can cause reproductive disorders.

**Objective:**

The effect of Naringenin (NG) on BPA-induced Sertoli cell toxicity and its mechanism was examined in the present study.

**Materials and Methods:**

In this experimental-laboratory study, the mouse TM4 cells were treated to BPA (0.8 μM) or NG for 24 hr at concentrations of 10, 20, and 50 μg/ml. Cell viability, reactive oxygen species (ROS) production, malondialdehyde (MDA) content, antioxidant level, and mitochondrial membrane potential (MMP) were examined. The expression of mitophagy-related genes, including Parkin and PTEN-induced putative kinase 1 (Pink1), was also evaluated.

**Results:**

BPA significantly lowered the viability of the Sertoli cells (p= 0.004). Pink1 and Parkin levels of the BPA group were significantly increased (p < 0.001), while the MMP was considerably decreased (p < 0.001). BPA raised MDA and ROS levels (p < 0.001) and reduced antioxidant biomarkers (p= 0.003). NG at the 20 and 50 μg/ml concentrations could significantly improve the viability and MMP of TM4 cells (p= 0.034). NG depending on concentration, could decrease Pink1 and Parkin at mRNA and protein levels compared to the BPA group (p = 0.024). NG enhanced antioxidant factors, while ROS and MDA levels were decreased in the BPA-exposed cells.

**Conclusion:**

The beneficial impacts of NG on BPA-exposed Sertoli cells are related to the suppression of mitophagy and the reduction of oxidative stress.

## 1. Introduction

Bisphenol A (BPA), an endocrine and estrogenic disrupting agent, is widely used as polycarbonate plastics and epoxy resins for producing food containers, water bottles, medical devices, and other objects that must be made of flexible and lasting materials. It can leak into food and drinks from plastic containers and accumulate in humans' bodies (1). BPA exposure at environmentally relevant concentrations can cause reproductive disorders (2).

BPA involves multiple mechanisms, including interference with mitochondrial functions (3). The toxicity of BPA relates to the excessive production of reactive oxygen species (ROS) that overwhelm the intracellular antioxidants (4). Mitochondria is the primary source of ROS, and under physiological conditions, its production and clearance are regulated by antioxidant and oxidation systems (5). BPA can induce mitochondrial dysfunction by lowering mitochondrial membrane potential (MMP) (6).

Autophagic removal of dysfunctional mitochondria (mitophagy) is activated in response to various toxic agents (7). The PTEN-induced putative kinase 1 (Pink1)/Parkin signaling pathway is the primary mechanism of mitophagy. Pink1 and Parkin play an essential role in the final checkpoint of mitophagy (8). Sertoli cells are targeted for various chemicals, and their damage indicates testicular toxicity (9). Several investigations have evidenced that BPA affects Sertoli cell functions (10). In an experimental study, BPA time- and dose-dependency reduced Sertoli cell viability (11). The study further revealed that Sertoli cells treated with BPA in vitro at a concentration of 200 μM induced morphological distortions such as collapsing of the cytoskeleton, chromatin impairment, and DNA damage in the cells.

BPA induces Sertoli cell death in rodents by causing mitochondrial dysfunction and excessive ROS generation (4). Feng et al. showed that BPA induces cell cycle arrest and apoptosis by preventing ROS levels in the porcine Sertoli cells (12). It is, therefore, necessary to find preventive and therapeutic agents to address BPA-induced reproductive toxicity.

Natural antioxidants potentially inhibit the adverse impacts of BPA on the reproductive system (13). Naringenin (NG) is a member of bioflavonoids derived from citrus species (14).

NG has several biological and pharmacological properties, such as anti-cancer, lipid reduction, superoxide elimination, anti-atherosclerosis, and antioxidants (15–19). It has been evidenced that NG displays antioxidant effects both in vivo and in vitro (19). The beneficial impacts of NG on the reproductive system have been reported in previous studies (20, 21).

In a previous study, NG could ameliorate lead acetate-induced reproductive toxicity and increase germ cell survival (22).

Due to the widespread use of BPA and its negative effects on the human reproductive system, it seems necessary to find a compound that ameliorates its negative effects. Based on the beneficial impacts of the NG on the male reproductive system, the present work has examined the NG's impacts on BPA-induced toxicity, mitophagy, and oxidative stress in TM4 cells (a mouse Sertoli cell line).

## 2. Materials and Methods

### Experimental design

In this experimental study, the TM4 cells were purchased from the Iranian National Center for Genetic and Biological Resources. This study was performed in 2023 at the Cellular and Molecular Research Center of Ahvaz Jundishapur University of Medical Sciences, Ahvaz, Iran. The cells were cultured at 37 C in 5% CO ${\;}_{2}$ in completed Dulbecco's Modified Eagle's Medium (DMEM, Merck, Germany) media. BPA and NG were dissolved in dimethyl sulfoxide (Sigma-Aldrich Chemie, Steinheim, Germany) and diluted in DMEM. The cells were treated with different concentrations of NG (Sigma-Aldrich, USA) and BPA (Sigma-Aldrich, USA). To explore the effect of NG on BPA-induced damage of TM4 cells, the cells were harvested in a medium containing various concentrations and times as a pilot study (Table I). The sublethal dose of BPA in TM4 cells was determined to be 0.8 μM. The protective dose of the NG was also identified based on a pilot study. The TM4 cells were divided into 4 groups:

• Control: received only culture medium for 24 hr.

• NG: received culture medium and 50 μg/ml NG for 24 hr.

• BPA: received culture medium and 0.8 μM/ml BPA for 24 hr.

• NG + BPA groups: received culture medium supplemented with BPA and NG at 10, 20, and 50 μg/ml concentrations for 24 hr.

### 3-[4,5-dimethylthiazol-2-yl] -2,5 diphenyl tetrazolium bromide (MTT) assay

The cells were cultured in a 96-well plate (10^4^ cells/well) for 24 hr. The media was discarded, and NG (25, 10, 20, 50, and 100 μg/ml) or BPA (0.8 μM/ml) for 24, 48, and 72 hr was added to indicate the concentration and duration efficacy. The cells were treated with an MTT (Sigma-Aldrich, USA) solution (0.5 mg/ml) for 3 hr. The supernatants were discarded, 100 μl dimethyl sulfoxide was added, and their absorbance was read at 570 nm.

### Measurement of intracellular ROS, malondialdehyde (MDA), and antioxidant levels 

ROS-producing was assessed by the florescent probe 2–7-dichlorofluorescein diacetate (DCFH-DA, Sigma-Aldrich, USA). After treatment, the medium was removed, and the ROS indicator DCFH-DA (10 μg/ml) in media was added and incubated for 20 min (37 C). After washing the cells with PBS, the ROS levels were detected with a spectrofluorometer (LS50B, USA; Em: 570 nm; Ex: 490 nm). After homogenization, the TM4 cells were lysed, and their proteins were determined using a BCA protein assay kit (Sigma-Aldrich, USA). The MDA, superoxide dismutase (SOD), catalase (CAT), glutathione peroxidase (GPx), and glutathione (GSH) levels were measured based on the kit's directions (ZellBio, Germany).

### Mitochondria isolation

The cells were re-suspended in an isolation buffer (250 mM sucrose, 20 mM HEPES-KOH, 10 mM KCl, 1.5 mM MgCl2, one mM EDTA, 1 mM EGTA, fat-free BSA 0.1%). The cells were homogenized and centrifuged (4 C) at 750 g for 10 min. The supernatants were again centrifuged (12,000 g) for 30 min. The pellets were then re-suspended in an isolation buffer.

### MMP evaluation

Fluorescent cationic dye Rhodamine 123 (Beyotime, China) was used for the MMP measurement. The TM4 cells, after treatment, washed with PBS twice and exposed to Rhodamine 123 solution (2.5 μg/ml) for 25 min at 37 C. A spectrophotometer (LS50B, USA; emission: 535 nm; excitation: 490 nm) was used to determine fluorescence.

### Real-time polymerase chain reaction

The RNeasy kit (Takara, Japan) was used to extract the RNAs from the TM4 cells (1 × 10^7^ cells), and cDNA was made by a cDNA synthesis kit (Takara, Japan). The cDNA was amplified in PCR reaction buffer (25 μL), including SYBR green and primers. A 45 cycle was used for PCR amplification: 95 C: 10 sec; 95 C: 15 sec; 55–57 C: 20 sec; 60 C: 20 sec. Normalization of the relative gene expression was done using the housekeeping *GAPDH* gene. Data were analyzed by the 2 ${\;}^{-\Delta \Delta \text{CT}}$ method. The following primers were used in this study: GAGACGATACCGACAAACAC (forward Pink1), GGCATTTCCTCCAAGACTAAC (reverse Pink1); TGCTCGTCAACCTCTGTTC (forward Parkin), TCACTTTCTCCTTCCCATCC (reverse Parkin).

### Measurement of Pink1 and Parkin protein levels

Pink1 and Parkin proteins were measured using ELISA kits (Biospes, China). Briefly, Pink1 and Parkin proteins of the mitochondrial fractions or cell lysates were bound to the primary antibodies and detected by a Horseradish peroxidase-conjugated secondary antibody. Quantification was done by recording the optical density at 450 nm. Pink1 sensitivity was more than 0.07 ng/ml in a range of 0.156–10 ng/ml, and Parkin sensitivity was 0.78 pg/ml in a range of 3.12–200 ng/ml.

**Table 1:** Viability percentage in TM4 cells at different doses and duration times of BPA treatment (pilot study)


**Treatments**	**12 hr**	**24 hr**	**48 hr**
**0 μM**	100	100	100
**0.1 μM**	98.1	95.3	92.5
**0.2 μM**	95.2	90.4	85.2
**0.4 μM**	91.4	84.5	67.5
**0.8 μM**	83.6	50.1	40.4
BPA: Bisphenol-A			

### Ethical considerations

The Ethics Committee on Animal Research of Ahvaz Jundishapur University of Medical Sciences, Ahvaz, Iran, approved the current work (IR.AJUMS.REC.1402.050).

### Statistical analysis

All statistical analyses were performed using the Statistical Package for the Social Sciences (SPSS) 21.0 software (SPSS, Chicago, IL, USA). One-way analysis of variance (ANOVA), followed by Tukey's or LSD tests, was used to analyze the data. P-value < 0.05 was regarded as statistically significant. All data were reported as mean ± SD. Kolmogorov-Simonov test was used for assessing the normal distribution of variables.

## 3. Results

### MTT assay 

**Figure 1:**
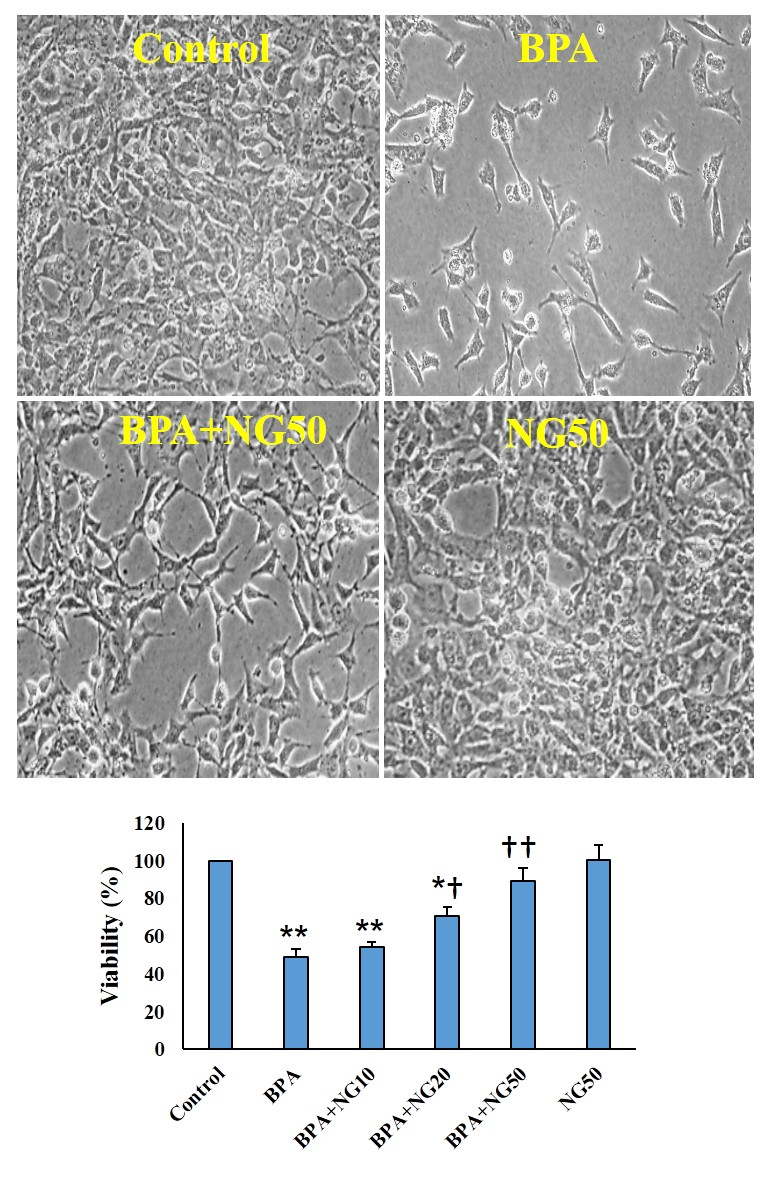
Viability of TM4 cells. Data are illustrated in Mean ± SD(n = 6). *P < 0.05, **P < 0.01, † P < 0.05, †† P < 0.01. Symbols indicate comparison to the control (*) and BPA ( † ) groups. BPA: Bisphenol-A, NG: Naringenin.

The survival of the NG-treated cells was not more remarkable than the control. In the BPA-exposed cells, the cell viability was lower than the control (p < 0.01). In the NG treatment at 10 μg/ml concentration, the viability percentage was slightly higher than in the BPA group, but it was insignificant. In the NG at 20 μg/ml and 50 μg/ml groups, the viability percentage was more significant than the BPA group (p < 0.05) (Figure 1).

### MDA, ROS, and antioxidant levels

**Figure 2:**
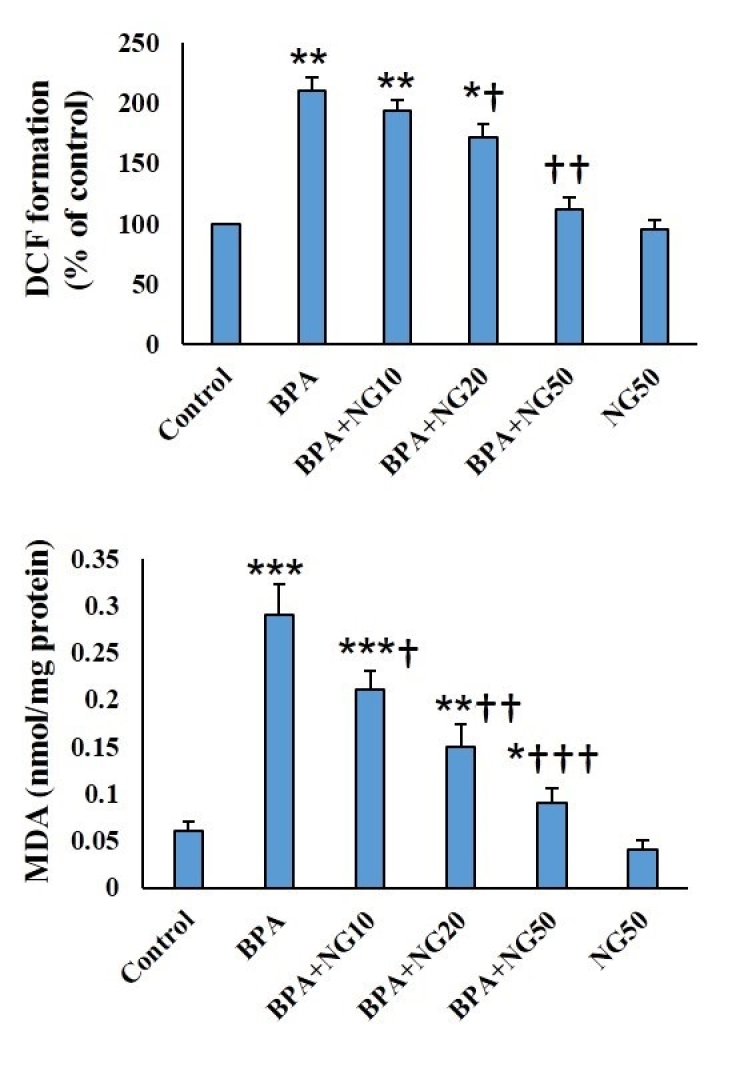
DCF (ROS) and MDA contents. Data are illustrated in Mean ± SD (n = 6). *P < 0.05, **P < 0.01, ***P < 0.001, † P < 0.05 †† P < 0.01, ††† P < 0.001. Symbols indicate comparison to the control (*) and BPA ( † ) groups. BPA: Bisphenol-A, NG: Naringenin.

**Figure 3:**
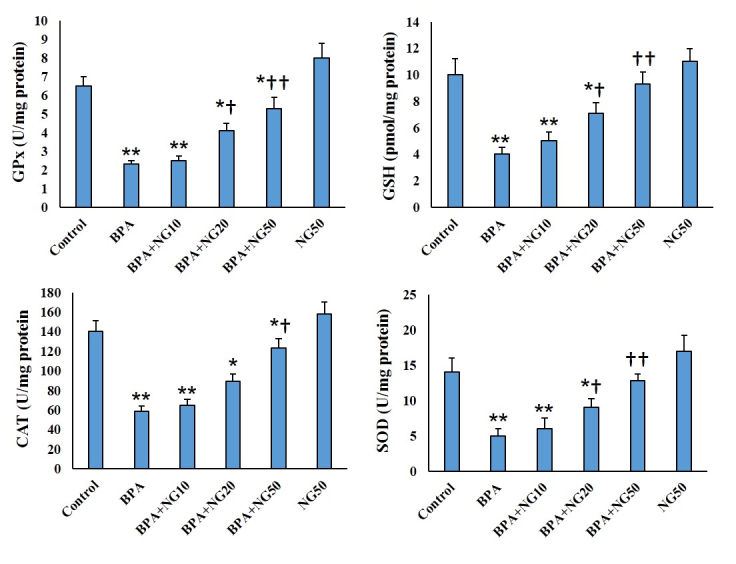
CAT, GSH, GPx, and SOD contents. Data are illustrated in Mean ± SD (n = 6). *P < 0.05, **P < 0.01, † P < 0.05, †† P < 0.01. Symbols indicate comparison to the control (*) and BPA ( † ) groups. BPA: Bisphenol-A, NG: Naringenin.

MDA and ROS amounts of BPA-exposed cells were more significant than their counterparts in the control group (p < 0.001). NG at all concentrations could significantly keep MDA and ROS levels lower than the BPA group. BPA administration significantly lowered the SOD, CAT, GPx, and GSH activity compared to the control (p < 0.01). NG concentration-dependently could enhance the SOD, CAT, GPx, and GSH activity compared to the BPA (Figures 2, 3).

### MMP assay 

**Figure 4:**
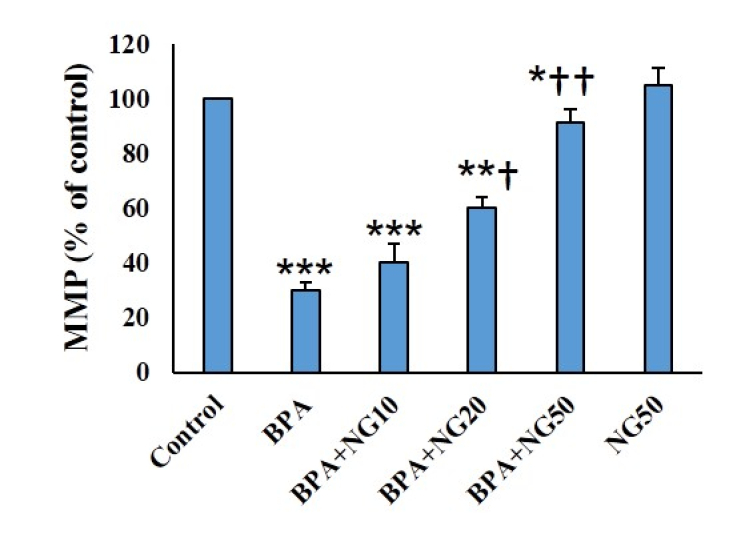
MMP measurement in different groups. Data are depicted in Mean ± SD (n = 6). *P < 0.05, **P < 0.01, ***P < 0.001, † P < 0.05, †† P < 0.01. Symbols indicate comparison to the control (*) and BPA ( † ) groups. BPA: Bisphenol-A, NG: Naringenin.

BPA significantly decreased the MMP of the TM4 cells compared with control (p < 0.001). NG, at the 20 μg/ml and 50 μg/ml concentrations, significantly enhanced the MMP compared to the BPA group (p < 0.05 and p < 0.01, respectively) (Figure 4).

### Real-time polymerase chain reaction

**Figure 5:**
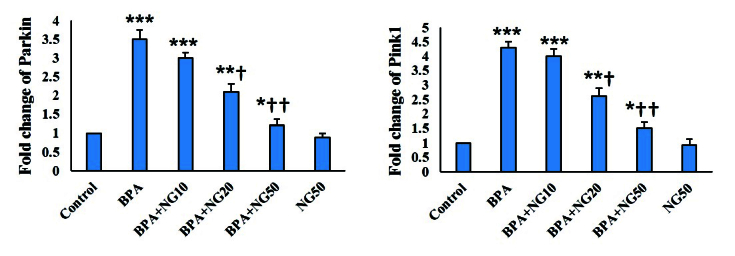
Pink1 and Parkin mRNA level. Data are depicted in Mean ± SD (n = 6). *P < 0.05, **P < 0.01, ***P < 0.001, † P < 0.05, †† P < 0.01. Symbols indicate comparison to the control (*) and BPA ( † ) groups. BPA: Bisphenol-A, NG: Naringenin.

Pink1 and Parkin mRNA levels of BPA-exposed cells were significantly higher than their counterparts in the control (p < 0.001). NG at the concentration of 20 μg/ml and 50 μg/ml could significantly reduce Pink1 and Parkin compared to the BPA group (p < 0.05). NG at 10 μg/ml concentration decreased Pink1 and Parkin levels compared to the BPA group, but it was insignificant (Figure 5).

### Pink1 and Parkin protein expression

**Figure 6:**
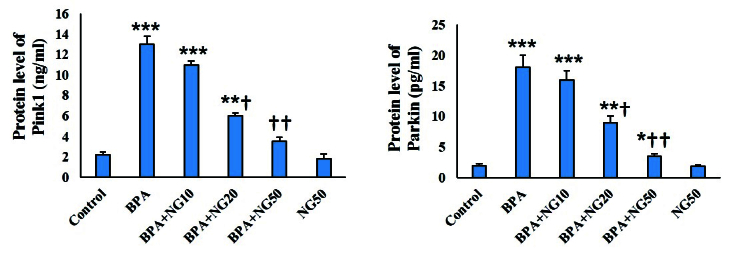
Pink1 and Parkin protein levels. Data are depicted in Mean ± SD (n = 6). *P < 0.05, **P < 0.01, ***P < 0.001, † P < 0.05, †† P < 0.01. Symbols indicate comparison to the control (*) and BPA ( † ) groups. BPA: Bisphenol-A, NG: Naringenin.

In the BPA-exposed TM4 cells, the Pink1 and Parkin protein levels were considerably higher than the control (p < 0.001). NG, at 20 μg/ml and 50 μg/ml concentration, significantly decreased the Pink1 and Parkin proteins compared to the BPA group (p < 0.05). NG at 10 μg/ml concentration did not affect the protein expression (Figure 6).

## 4. Discussion 

In the present study, we show the protective potential of NG against BPA-induced TM4 cell toxicity. BPA induced cytotoxicity by decreasing cell viability, enhancing ROS production, stimulating mitophagy, and disrupting mitochondria in the Sertoli cells. The reduced viability demonstrated the toxic effects of BPA on the TM4 Sertoli cells. In line with our results, a previous study showed that BPA reduced the survival rate of the TM4 cells (23).

NG dose-dependently reversed the viability of the BPA-exposed cells. In parallel to our findings, have reported the protective impacts of NG against permethrin-induced testicular toxicity in rats (24). Have demonstrated that NG improves some reproductive parameters, apoptosis, and oxidative stress in acrylamide-induced testis toxicity of rats (25).

The present study showed that BPA increases oxidative stress in TM4 cells, manifested by rising MDA and ROS levels and decreasing antioxidant GSH, GPx, SOD, and CAT levels. The present observation agrees with previous findings showing an increase in MDA level and a decrease in the antioxidant activity in BPA-induced TM4 cell toxicity (26).

NG ameliorated BPA toxicity by reducing the elevated MDA and ROS levels and increased GSH, GPx, SOD, and CAT levels. Previous studies indicated that NG has potent antioxidant activity (13, 18, 21). For example, Mehranfard et al. mentioned that NG has a protective effect on gentamycin/antibiotic-induced testicular oxidative stress (21). Accordingly, NG may have a protective effect on BPA-induced toxicity by modulating the antioxidant system. In this study, BPA significantly diminished the MMP of the TM4 cells, and NG could concentration-dependently reverse the MMP level. BPA resulted in a loss of MMP in goat Sertoli cells (23). BPA causes hepatotoxicity by inducing mitochondrial disruption in rats (3).

Dysfunctional mitochondria are cleared using the mitophagy process (27). Hence, we explored the impact of BPA on the Parkin and Pink1 levels, which are involved in mitochondrial quality control (28). BPA enhanced the protein level of Pink1 and Parkin in mitochondrial fractions. Anand et al. evidenced that BPA stimulates mitophagy by the accumulation of Pink1 and translocation of Parkin to damaged mitochondria of primary rat hepatocytes (29). The reduction of MMP was associated with BPA-induced cell death (30). Overexpression of Parkin induces mitochondria removal by mitophagy when MMP is depleted (8). The enhanced expression of Pink1 and Parkin proteins indicates that reducing Sertoli cell viability by BPA is due to the mitophagy stimulation.

The mitophagy process closely relates to cell death (31, 32). The abnormal excessive mitophagy eliminates mitochondria, and cells lose their essential functions and undergo death (33, 34). In high-toxicity conditions, mitochondria cannot be repaired by mitophagy (35). Overexpression of Pink1 activates mitophagy and inhibits apoptosis (36).

In a previous study, Pink1/Parkin-mediated mitophagy could inhibit apoptosis in osteoblasts (37). Wei et al. showed that naringin (another bioactive polyphenol in citrus fruits) decreased the inflammatory response, oxidative stress, and apoptosis via regulating endoplasmic reticulum stress and mitophagy in a mouse model of pulmonary fibrosis (38). Feng et al. showed that naringin reduced cerebral ischemia-reperfusion damage in rats through mitophagy activation. In their study, naringin could suppress apoptosis and inhibit the translocation of Parkin to the mitochondria in the ischemia-reperfused rat brains (12). Conversely, excessive mitophagy induces apoptosis in cancerous cells, in particular (39). Chen et al. showed that ketoconazole encourages mitophagy to induce apoptosis in hepatocellular carcinoma. In another study, pardaxin induced excessive mitophagy and apoptosis in human ovarian cancer cells (40).

Our results showed that NG concentration-dependently reduced Pink1 and Parkin expression. Therefore, NG can reduce mitochondria dysfunction and mitophagy induced by BPA.

## 5. Conclusion

This study demonstrated that exposure to BPA raised Pink1 and Parkin levels in TM4 cells, which increased mitochondrial dysfunction and mitophagy. This study has also revealed that NG attenuates the cytotoxic impacts of BPA on the TM4 Sertoli cells. NG effectively increased the activity of antioxidant enzymes and the production of ROS in cells treated with BPA. NG inhibited mitophagy by reducing the expression of Pink1 and Parkin while simultaneously enhancing MMP in the exposed cells to BPA.

##  Data availability

Data supporting the findings of this study are available upon reasonable request from the corresponding author.

##  Author contributions

YAF and LKh devised the project, the main conceptual ideas, and proof outline. EY and AHM monitored, evaluated, and analyzed the results of the study. Further, YAF and MJKh wrote the manuscript with support from LKh. All authors discussed the results and approved the final manuscript and took responsibility for the integrity of the data.

##  Conflict of Interest

The authors declare that there is no conflict of interest.
